# Linking household and facility data for better coverage measures in reproductive, maternal, newborn, and child health care: systematic review

**DOI:** 10.7189/jogh.06.020501

**Published:** 2016-12

**Authors:** Mai Do, Angela Micah, Luciana Brondi, Harry Campbell, Tanya Marchant, Thomas Eisele, Melinda Munos

**Affiliations:** 1 Department of Global Community Health and Behavioral Sciences, Tulane University School of Public Health and Tropical Medicine, Tulane, LA, USA; 2 Department of Global Health Management and Policy, Tulane University School of Public Health and Tropical Medicine, Tulane, LA, USA; 3 Centre for Population Health Sciences, The University of Edinburgh, Edinburgh, Scotland, UK; 4 Faculty of Infectious and Tropical Disease, London School of Hygiene and Tropical Medicine, London, UK; 5 Center for Applied Malaria Research and Evaluation, Department of Tropical Medicine, Tulane University School of Tropical Medicine, Tulane, LA, USA; 6 Department of International Health, Johns Hopkins Bloomberg School of Public Health, Baltimore, MD, USA

## Abstract

**Background:**

Currently many measures of intervention coverage obtained from household surveys do not measure actual health intervention/service delivery, resulting in a need for linking reports of care–seeking with assessments of the service environment in order to improve measurements. This systematic review aims to identify evidence of different methods used to link household surveys and service provision assessments, with a focus on reproductive, maternal, newborn and child health care, in low– and middle–income countries.

**Methods:**

Using pre–defined search terms, articles published in peer–reviewed journals and the grey literature after 1990 were identified, their reference lists scanned and linking methods synthesized.

**Findings:**

A total of 59 articles and conference presentations were carefully reviewed and categorized into two groups based on the linking method used: 1) indirect/ecological linking that included studies in which health care–seeking behavior was linked to all or the nearest facilities or providers of certain types within a geographical area, and 2) direct linking/exact matching where individuals were linked with the exact provider or facility where they sought care. The former approach was employed in 51 of 59 included studies, and was particularly common among studies that were based on independent sources of household and facility data that were nationally representative. Only eight of the 59 reviewed studies employed direct linking methods, which were typically done at the sub–national level (eg, district level) and often in rural areas, where the number of providers was more limited compared to urban areas.

**Conclusions:**

Different linking methods have been reported in the literature, each category has its own set of advantages and limitations, in terms of both methodology and practicality for scale–up. Future studies that link household and provider/facility data should also take into account factors such as sources of data, the timing of surveys, the temporality of data points, the type of services and interventions, and the scale of the study in order to produce valid and reliable results.

Access to quality health care is critical in order to ensure better population health outcomes in areas like maternal and child health. Throughout the MDG era, increases in access to services have been observed, but improvement in population health outcomes has not been consistently documented [1–4]. Consequently, much effort has been put towards improving population access to health care; yet adequately measuring the quality of care received remains challenging [5]. Many measures of coverage obtained from household surveys only estimate service contact (eg, coverage of antenatal care) instead of actual service delivery. Even when surveys do attempt to measure content of care (for example, for sick children), this measurement can be inaccurate [6,7]. In many cases, care–seekers are not able to reliably recall or report on different aspects of the quality of care in household surveys [8]; yet this information is important for several reasons. First, in order to improve population health outcomes a minimum level of quality of care must be guaranteed at the point of care. Second, it gives a more comprehensive assessment of the provider–client interaction and allows gaps in the quality of care to be assessed and improved upon. Third, for health planning and program evaluation purposes, it is necessary to measure the proportion of the population that actually receive an intervention with adequate quality.

In response, methods linking household data on care–seeking or service contact to health provider assessment data on service readiness or quality have emerged as a potentially effective strategy for improving coverage measurement. A growing number of studies have employed different linking approaches to either examine associations between the service environment and care–seeking behavior, or seek to improve coverage measures of health interventions. We conducted a systematic review of the literature to document different methods used to link household surveys and service provision assessments in low– and middle–income countries. We also investigated the feasibility, as well as methodological and practical advantages and limitations of the linking methods employed. The primary focus of the review was on reproductive, maternal, neonatal, and child health interventions.

## METHODS

### Search strategy and inclusion criteria

We conducted the literature search using a combination of search terms (**Table 1**), and scanning of reference lists of identified papers. The search was based on the following published databases: PubMed, Medline, JSTOR, Google Scholar, LILACS, and Population Health Metrics, which is a specialist online journal on this topic. Within each database, we used a combination of search terms (eg, “maternal health, service use, link, access to care”), changing one search term at a time. We also did a hand search of the grey literature on websites of the WHO, MEASURE Evaluation project, the Demographic and Health Survey (DHS) program, the World Bank, Carolina Population Center at UNC (UNC/CPC), and Google. Only reports and articles produced in 2004 or later were available on the UNC/CPC website. The search was conducted in English, Spanish, and French. Although the focus of the review was reproductive, maternal, neonatal and child health, we included a few relevant studies examining primary and curative care as they were identified during the search.

**Table 1:** Search terms that were used in the systematic search

Topic area	Household/Population–based data	Connection	Facility–based assessment
[null]	[null]	Link	Access to care
Maternal health	Service use	Linkage	Service quality
Antenatal care	Service utilization	Match	Quality of care
Postnatal care	Help seeking	Combine(d)	Service readiness
Delivery	Care seeking	Merge	Service provision
Childbirth	Doctor visit	Attach	Service delivery
Reproductive health	Clinic visit	Join	Where care was sought
Obstetric care	Facility visit	Pair	Facility survey/data/assessment
Women’s health service	Household survey/data/assessment	Connect	
Pregnancy complications			
Postpartum care			
Neonatal care			
Newborn care			
Child health care			
Child immunization			
Sick child visits			
Well child visits			
Family planning			
Contraception			

In order to be included in this review, a study had to meet the following criteria: 1) it was conducted from 1990 to March 2015, because of rapid development of maternal health care since the 1990s; 2) the study was in a low– or middle–income country; 3) household care–seeking information was linked with facility or provider characteristics; and 4) the study addressed coverage of interventions in the above mentioned areas.

### Definitions

We employed the WHO’s definition of coverage which is “coverage of health services can be measured by the percentage of people receiving the services they need” [9]. An intervention coverage indicator would be calculated based on the number of individuals in need of a particular service or intervention (ie, the denominator) and the number of individuals in need who are using or receiving the services (ie, the numerator). It is also important to note that in this review we used “service/intervention coverage” interchangeably with “health care–seeking behavior” since the latter seemed more common in the literature and we were primarily interested in methodologies used to link service and provider’s characteristics with individual care–seeking rather than the actual content of health care service or intervention.

## RESULTS

**Figure 1:**
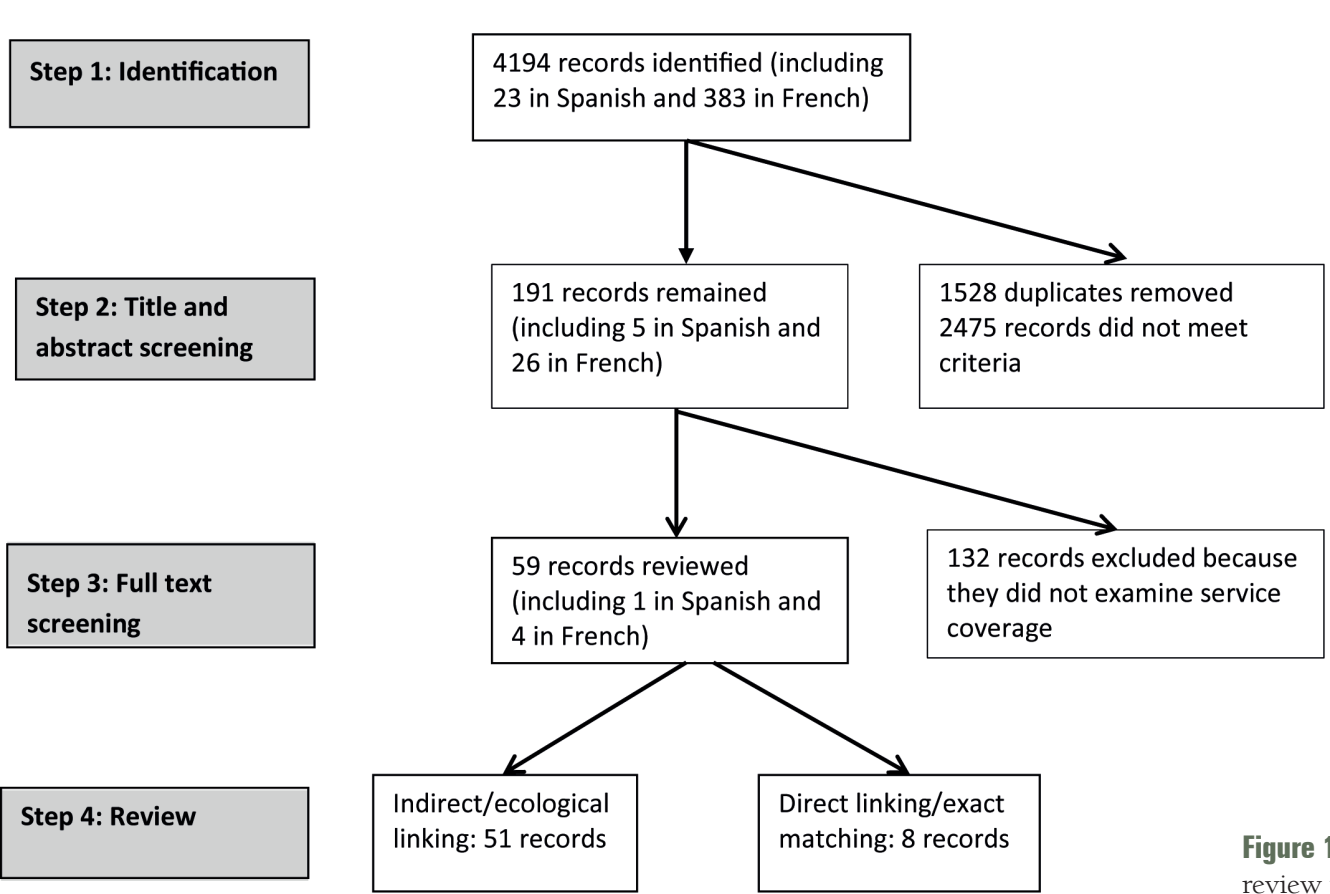
Flowchart of the review process.

**Figure 1** shows the number of articles identified after each step of the literature search. The initial number (n = 4194) included a number of articles that turned up in more than one database. After removing 1528 duplicates, screenings of the title and abstract removed the majority of the articles (n = 2475), because the studies presented either employed only one source of data (either household or facility). Further full–text screening of the remaining 191 articles resulted in the exclusion of about two–thirds of them because the articles did not examine coverage or service characteristics at the provider or facility level. The remaining articles and conference presentations (n = 59) were carefully reviewed and categorized into two groups based on the linking method used: 1) indirect/ecological linking that included studies in which health care–seeking behavior was linked to all or the nearest facilities or providers of certain types within a geographical area, and 2) direct linking/exact matching where individuals were linked with the exact provider or facility where they sought care. These articles were summarized in Table S1 in the **Online Supplementary Document(Online Supplementary Document)**.

Among the studies reviewed, 13 were published in the 1990s; the rest after 2000. The studies were mostly from Asia and Africa, seven were from Latin America and the Caribbean [10–16]. Many (n = 35) studies were conducted in rural areas of a country or limited to an administrative sub–national region (state or province). Care–seeking behaviors also varied: from curative care for sick adults to family planning, maternal and child care, malaria and HIV related services; they also varied from care–seeking that does not always require visits to a health facility, such as FP knowledge, intention, and use, to interventions that are by default facility based, such as institutional delivery.

### Linking approach – indirect (ecological) linking

This approach was employed in 51 of 59 included studies, and was particularly common among studies that were based on independent sources of household and facility data like the DHS and Service Provision Assessment (SPA) [17]. With this approach, surveyed households and individuals were often linked to all or the nearest providers of certain types within a geographical area, eg, cluster, local government areas (LGA), local council areas, region or zone. In other words, health seeking behaviors reported in the household survey in a region were linked to provider data that had been aggregated to the same region level [18–20]. The higher level of geographical areas (LGA, region, or zone) was often used if there were a small number of providers within a cluster, or if there were concerns of the representativeness of providers at the lower level [21]. For example, the SPA is not designed to provide representative results on facilities at the cluster level; as a result, in studies that used nationally representative data like the DHS and SPA, provider data for linking were often aggregated to the region or zone level rather than the cluster level. Consequently, surveyed households were not necessarily attached to measures of intervention at the providers from which household member might have realistically sought care.

Linking from households to providers or facilities within a geographical area could also be done using the cluster/area identification where one or many providers within the area were linked to each household in the same area. In some cases, linking was done administratively between households and providers designated to serve each village or cluster [5,22–28] or to the one most frequently used [29]. In other cases, boundaries of the geographical area were established and each household was linked to providers/facilities within their cluster/geographical area and possibly with providers within the neighboring clusters [30]. In the latter, GPS coordinates were often used to establish geographical distances from each household to each of the connected providers [16,31–35]. In general, distances could be calculated as a straight–line distance or travel distance and travel time using the most convenient road(s) as reported by the households or key community members [1,33,36–41]. One study provided a detailed review of four geographical techniques often used to link household clusters with facilities [42]: 1) administrative boundary link, 2) Euclidean buffer link, 3) road network link, and 4) Kernel density estimation link (**Box 1**).

Box 1 **Geographical methods used to link household surveys and assessments of service (Skiles, 2013) [41]**

Administrative boundary link: health facilities are linked to DHS clusters within the same administrative limit (eg, district).

Euclidean buffer link: each DHS cluster is the center of a 5 km Euclidean buffer (the 5km Euclidean buffer is an approximation for a 1–hour walking maximum distance between the DHS cluster and the health facility). Each cluster is then linked to each facility within this buffer and administrative boundaries are not considered.

Road network link: uses the road network to calculate the distance between each cluster and a facility (only a total distance of less than 15 km between a cluster and a facility is considered a link). The distance from each cluster or each facility to the road should be less than 5 km.

Kernel density estimation (KDE) link: this is a fairly sophisticated GIS–based spatial analysis technique used to distribute a value associated with a discrete point across a plane or continuous surface. This technique assumes that each facility serves a specific catchment area and that the draw on the population to those services decreases with increasing distance from the facility. This “draw” of each facility varies according to the type, size, and availability of services. Therefore, with this technique, it is possible to incorporate facility characteristics and distance decay when estimating the potential draw a facility may have on a population cluster.

In a small set (n = 11) of studies in the indirect linking group, physical accessibility was the only characteristic of the service environment measured and linked with household data on care–seeking; no provider assessment was conducted. Each surveyed household was connected with one or more nearest health facilities using measures of physical accessibility, regardless of whether they sought care at these facilities [23,24,33,35,43–48]. Physical accessibility was often measured by straight–line distance, driving distance, and walking or driving time.

**Data sources.** In most studies, two independent sources of household and facility data were used. For example, 16 out of 46 studies employed DHS household data, combined with a SPA (or its predecessor Service Availability Module SAM) or a situation analysis [49–54]. In these cases, the scope of the study was usually at the national level or limited to rural areas. Few studies employed data from a population census and a facility census–either at the national level (Zambia) [55] or the district level (Burkina Faso) [22]. The other studies often employed data from household and facility surveys that were conducted as part of a larger project, such as COMPASS (Community Participation for Action in the Social Sectors) in Nigeria [21] or DISH (Delivery of Improved Services for Health) in Uganda [30].

With this type of linking, the proportion of individual reporting care–seeking can be obtained from the household survey, and it may be possible to calculate the percentage of providers who provide a specific intervention. The measure of coverage, however, may be more useful at the population level than at the individual level as each individual is linked to an aggregate measure of service environment. Additionally, if the health intervention of interest was often utilized by the population in a facility’s catchment area, it would be reasonable to assume that measures of readiness and quality of care, when aggregated to the facility level, represent the level of care that surveyed individuals received.

The time interval between the household survey and the linked provider/facility assessment varied between studies and by the type of intervention: it ranged from current use of FP to child vaccination of children up to 10 years of age [33]. In the majority of the studies, they were conducted within two years. If they were part of a larger project, like in COMPASS or DISH, they were likely conducted within the same year. In some cases, particularly for studies that relied on secondary data like the DHS and SPA, the gap could be longer: four to five years [19,55–57]; yet because of the 3–5 year recall period often used in the DHS, the actual gap between care–seeking and provider assessment could be shorter if the DHS was linked to an earlier SPA. It is important to note that even when the surveys were conducted in the same year, recall periods in the household survey (eg, antenatal care sought for live births in the five years prior to the survey) meant that the actual gap between measured care–seeking behavior and service characteristics was often wider.

**Limitations.** Limitations of the indirect linking approach were not discussed in all of the studies reviewed but we have identified the following limitations from the different approaches used. A major limitation was that the linked facilities/providers may not be ones that surveyed individuals sought care from as bypassing of facilities is a common phenomenon [32]. Although also applied to direct linking, the time interval between surveys was mentioned as a limitation in linking in several studies as many characteristics of the service environment, eg, supply and medicine stock–outs may change rapidly over time [15,16,42,56,57]. Another limitation was that the surveyed facilities may not represent the entire market of services that individuals can choose from [21,22,57–59]; this was particularly important for interventions like FP, ANC, child immunization, etc. as individuals can obtain the intervention from providers outside of the formal health sector and therefore not included in most service assessment. In addition, administrative linking using cluster identification may also be susceptible to errors due to mis–identification and displacement of cluster and cluster boundaries [12,60]. Finally, several limitations related to the use of geographical distances were mentioned, including that straight–line distances did not take into account differences in terrains and transports [15,38,55,56].

### Linking approach – direct linking (exact matching)

Only eight of the 59 studies included in this review employed direct linking methods. In this case, individuals were linked with the exact provider or facility where they sought care from. This type of matching was typically done at the sub–national level (eg, district level) and often in rural areas, where the number of providers is more limited compared to urban areas. The type of health services/interventions varied, from sick care for adults or children to child vaccination, and delivery care. On the service provision side, a number of measures of service availability, access (including physical access, hours of operation), and readiness (availability of drugs, equipment, trained providers) were used. Studies did not always assess the actual quality of care that individuals interviewed received in the past. Instead, provider–client interactions were observed on a separate sample of clients, independent of those interviewed in the household survey. A necessary assumption is that the quality of care does not substantially change during the period between the household and the facility surveys.

Although two sources of data were typically used, the sequencing of data collection varied between studies. In the first approach, data were first collected on readiness and/or service quality from clinics, then facility records were used to identify patients (adults or children) who would then be followed up at home. This approach was employed in four studies [61–64]. A limitation of this approach was the possibility of self–selection bias amongst care–seekers, which means that those who sought care at these providers were different from those who did not seek care or sought care elsewhere in many characteristics. Another limitation is the potential underestimation of some indicators; for example, one study [63] reported that even if facility records showed that some children missed immunization shots, they might have received the shots elsewhere as families could move around. Consequently, this data cannot be used to produce estimates of coverage at the population level.

In three studies [13,65,66], the opposite approach was adopted: individuals who sought care were first asked for the names of specific facilities from which they sought care and these facilities were subsequently surveyed. For instance, in the Ghana study, women of childbearing age in a demographic surveillance district were matched with health facilities where they reported having received delivery and post–partum care for all live births during a one year period; the data were then linked with a census of all health facilities within the district [65]. Similarly, in the Kenya study, women were linked to the facility that they reported having received services from last [66]. Another study [67] employed a similar approach but using existing data: all children under five at a demographic surveillance site were linked to clinic visits using a unique identification number. An apparent strength of the Ghana study relative to the others was that data were collected from all live births and all health facilities in the district [65,67]. On the other hand, the use of a demographic surveillance site in these two studies has implications for the replicability of the approach.

## DISCUSSION

There is a growing body of research in which household survey data are linked with provider assessments: 59 articles have been published in peer–reviewed journals or in the grey literature since 1990. It is noteworthy that most of the reviewed studies aimed to examine the associations between service environment characteristics and care–seeking behavior at the individual or household level rather than trying to better understand intervention coverage, ie, the proportion of individuals in need of an intervention who actually receive it with adequate quality. These linked study designs present a number of complex methodological issues, which we discuss below with particular attention to how these issues might affect the use of linking designs to estimate intervention coverage.

This review highlights two major linking methodologies: indirect/ecological linking and direct linking/exact matching. Most studies that sought to link household survey and service provision data used indirect or ecological linking, generally using two independently collected and sampled data sources linked at national level. We found eight studies that employed direct linking or exact matching. Unlike for indirect linking, these studies were generally conducted at sub–national level, often in rural settings where the provider mix was less complex. In addition, the data sources used for direct linking were not independent.

These two linking approaches have trade–offs in terms of ease of implementation and usability of the data. Indirect linking appears less expensive and simpler to use than direct linking. In most cases, the indirect linking studies used two independent samples of households and facilities, such that both samples could be designed to be representative of a geographic area. Cautions need to be exercised, however, if one is to use nationally representative data like the DHS and SPA, as these surveys are often not designed to be representative at a level lower than region. Independent sampling also simplifies implementation, as the sampling for one survey does not depend on the other. However, one drawback of this approach is that, since the surveys are sampled independently, it is possible that households may be linked to providers that are not representative of the providers used by the household. A second limitation is related to bypassing of facilities, meaning that individuals do not always seek care from the nearest provider or one that is designated to serve the area, and in fact may travel quite a distance to a provider that is perceived to provide better quality of care. If geographical linking of individuals to the nearest providers is used in a setting where bypassing is prevalent, the results may be invalid. For studies that use DHS data, in which there is geographical displacement of clusters, linking of individuals to the nearest providers may also produce invalid results; instead linking by administrative boundary methods may be less affected by the displacement [42]. In general, there is a need for further validation of indirect linking methods as compared to direct linking, particularly as relates to coverage measurement. While we assume here that ecological linking is less likely to produce valid results than direct linking, there have been no head–to–head comparisons of the two methods in the same population.

Unlike indirect linking, the objective of direct linking is explicitly to link an individual to his/her actual source of care. Thus, many of the limitations of indirect linking do not apply to direct linking. However, this approach has a number of limitations related to sampling. In this review we saw two approaches to linked sampling: either households were sampled from registers at health facilities, or else health providers were sampled based on sources of care reported by households. The first case would yield a sample of households that is not representative of the general population, meaning that this approach cannot be used for estimating population–based intervention coverage. The second case would yield a sample of providers that is not representative of the universe of providers, but would allow for population–based measures, as households are sampled to be representative of the population. In either case, the requirement to link the sampling for the two surveys is likely to complicate data collection.

Although linking is potentially a promising approach for estimating intervention coverage, it cannot be used for all interventions. In order for linking to be useful, it must be possible to measure care–seeking for the intervention through a household survey, and the intervention must be delivered through a recognized provider that can be sampled. If the intervention does not always require a visit to an identifiable provider, this method may not be useful. For example, family planning users do not have to go to a clinic or even a pharmacy to obtain condoms or oral pills. In many settings, self–treatment for a sick child or adult may be common, and treatments may be obtained from shops and informal vendors in addition to pharmacies. In other words, researchers need to ensure that sampled health providers are representative and inclusive of different types of possible providers of the intervention.

An important component of measuring care–seeking in household surveys is correctly identifying the denominator, ie, the individuals in need of the service or intervention. Depending on the intervention, need may be defined based on age, sex, or pregnancy status, or may require the respondent to accurately report on symptoms of disease, such as fever or diarrhea. In the included studies, service needs were not explicitly defined; rather it was implicit using criteria like age groups (eg, children under five, women of reproductive age) or life stage (eg, pregnant women). In included studies examining care–seeking for sick adults or children, service needs were self–perceived, based on household members’ report of fever and other symptoms. Measurement of care–seeking for treatment of disease may be biased due to differences in respondents’ perceptions of illness and their ability to recognize, recall, and report symptoms.

It is unclear whether the respondents in household surveys are able to accurately report on whether care was sought, and if so, from which type of facility or cadre of provider. A recent study noted challenges in identifying the type of providers using DHS–type questionnaires due to respondents’ knowledge of source of care and the five year reference period used by DHS for these questions [8]. For example, if delivery care takes place at home, it may be difficult for the respondents to identify if the caregiver is from the public or private sector. Similarly, providers from the non–profit sector may not be easily identified by respondents if they are not well branded or if they work through the public or private sector. Valid measurement of care–seeking, including the type of provider or facility visited, is essential if we want to estimate intervention coverage using a linking approach, and therefore more data are needed on the validity of respondents’ categorization of sources of care.

This review highlights a few issues to consider when using a linking method to estimate intervention coverage. In most cases, it is not possible to measure service quality, readiness or what actually happens during service delivery to those who sought care. It is therefore assumed that measures of the service environment at the time of facility data collection are comparable to the (unmeasured) service characteristics at the time that care was sought. Meanwhile the service environment (availability, readiness and quality) may change rapidly because of changes in policy, funding, and development or quality improvement programs. In addition, factors like drug stock–outs are time–variable and can substantially impede the ability of a facility to provide quality care. It is therefore important that the time gap is minimized to reduce measurement errors. This time restriction may be a barrier especially for linked coverage measures of maternal and newborn interventions, since the reference period typically used in household surveys for collecting data on maternal and newborn care seeking behavior can be as much as five years prior to the survey. It is unlikely that the service provider data collected at a single point in time would be relevant to an entire 5–year period. It might therefore be desirable to conduct the service assessment within a short interval (eg, 12 months) of the household survey. Since recall of care–seeking in household surveys is retrospective, it will likely also be important to ensure that the service assessment is conducted before the household survey, in order to minimize the interval between when an intervention was received (and care–seeking was reported in the household survey) and when the quality of service at a provider was actually measured.

Finally, it is important to note that compared to surveys like MICS and DHS, which include urban and rural areas, linking studies that include only or primarily rural areas may be simpler and produce more valid results with regard to the service environment because the universe of health providers/facilities and their catchment population are easier to define. For this reason, some linking methods may be more appropriate to rural than urban and vice versa. For example, administrative boundary linking may work well in rural, but GPS–based physical distances may be more valid in urban settings. Further research is needed to understand the validity of various linking methods in different contexts.

In conclusion, several different methods linking care–seeking data from household surveys to readiness or service quality data from provider assessments have been employed in a growing body of research on health intervention coverage and can be classified into two broad categories: indirect linking and direct linking/exact matching. Each has their own advantages and limitations, in terms of both methodology and practicality. Future studies that aim to link household and provider data should also take into account important factors such as the timing of surveys and temporality of data points, the type of service and intervention, and the scale of the study in order to produce valid and reliable results. There is also a need for additional data on the validity of different linking approaches and the validity of care–seeking as reported in household surveys in order to inform development of these methods.
