# Supplementary material for: Linking household and facility data for better coverage measures in reproductive, maternal, newborn, and child health care: systematic review
Source: J Glob Health. 2016 Sep 3;6(2):020501. doi: 10.7189/jogh.06.020501 (PMC5012234; doi:10.7189/jogh.06.020501)
Supplement: Online Supplementary Document [file jogh-06-020501-s001.pdf]

Online Supplementary Document

Do et al. Linking household and facility data for better coverage measures in reproductive, maternal, newborn, and child health care: systematic review

J Glob Health 2016;6:020501

Table S1. Summary of included studies

No.	Authors and Year	Study Site	Level of linking	Source and Timing of Household Data	Source and Timing of Facility Data	Outcome
Indirect (Ecological) Linking						
1.	Acharya and Cleland, 2000	Nepal (western and middle western hill region)	*Cluster* level; used a subset of clusters in household survey and had to have a health post within 3 hours walking distance.	1991 Nepal Fertility, Family Planning and Health Survey	Supplementary Health Facility survey (56 clusters, 28 health posts) in 1994	Maternal and child health
2.	Akin et al., 1998	Sri Lanka	GIS-based spatial analysis at *household* level: each household was linked to the three closest facilities of each type and distance variables were created	Survey of ~10,000 households in 4 project areas in 1992	Survey of 781 health facilities in major public (i.e. hospitals), minor public (i.e. other types of facilities), and private sectors in 1992	Demand for curative health care

No.	Authors and Year	Study Site	Level of linking	Source and Timing of Household Data	Source and Timing of Facility Data	Outcome
3.	Akin and Hutchinson, 1999	Sri Lanka (Matale district)	GIS-based spatial analysis at *household* level: each household was linked to the three closest facilities of each type and distance variables were created	Survey of 1,672 households in the district in 1992	Survey of 314 health facilities in the district in 1992	Choice of facility for curative care
4.	Al-Taiar et al., 2010	Yemen (Taiz province)	*Household* level: 1) driving distance recorded by taking the shortest and most convenient road; 2) driving time; 3) GPS to calculate straight-line distance using Haversine formula[1]	Household survey at the home of children 6 months – 10 years old. Timing of survey was not reported.	Physical accessibility only.	Child vaccination
5.	Ali, 2001	Egypt (rural)	*Household* level: women were linked to service environment measures aggregated to the cluster level	1988 DHS	1989 Service Availability Survey	Contraceptive discontinuation
6.	Angeles et al., 2005	Peru	*Cluster* level: women were linked to oldest SDP of each of the 4 types (health centers, dispensaries, CBDs,	1991 DHS of 15,882 women in 901 clusters	1992 Situation Analysis: census of SDPs within 5km of	Fertility

No.	Authors and Year	Study Site	Level of linking	Source and Timing of Household Data	Source and Timing of Facility Data	Outcome
			and pharmacies)		each cluster	
7.	Benova et al., 2014	Tanzania	Provider information was aggregated to the level of *geographic zones* (8 zones: Central, Western, Lake, Southern Highlands, Southern, Northern, Zanziba, and Eastern	2010 DHS	2006 SPA	Water and sanitation environment of home- vs. facility-based childbirths
8.	Buor, 2005	Ghana (Ahafo-Ano South district and Kumasi metropolis)	*Household* level: distance and travel time to health facility	Sample of 390 women surveyed in 2000-2001	Physical accessibility only.	Utilization of curative health service
9.	Cronin, Guilkey and Speizer (unpublished)	Senegal (urban only)	*Household* level: link women to facilities within 1km of the PSU, GPS based	2011 baseline survey of 9,614 women from 6 sites	2011 census of 205 FP facilities and pharmacies from the same 6 sites	FP
10.	Dietrich et	Tanzania	*Cluster* level	1996 Demographic	1996 Tanzania Service	Fertility, FP, ANC, delivery,

No.	Authors and Year	Study Site	Level of linking	Source and Timing of Household<br><br>Data	Source and Timing of Facility Data	Outcome
	al., 1998			and Health Survey	Availability Survey<br><br>(1991 and 1994 surveys used as supplements as necessary)	child immunization, and HIV risk behaviors
11.	Do and Koenig, 2007	Vietnam	*Cluster* level	1997 Demographic and Health Survey - Household module	1997 Demographic and Health Survey – Health facility module	First-method discontinuation of contraceptive, all-method discontinuation of contraceptive
12.	Entwisle et al., 1997	Thailand (Nang Rong)	*Household* level: 1) spatial network analysis to estimate travel time associated with each of 4 types of road and a combination of road, and 2) Euclidean distance	Household census conducted in 1984	Physical accessibility only.	Contraceptive choice
13.	Faye et al., 2010 (in	Senegal	*Household* level: distance to health facility	Survey in 2007 of women who gave birth July 2006-June 2007	Physical accessibility only.	Home birth

No.	Authors and Year	Study Site	Level of linking	Source and Timing of Household Data	Source and Timing of Facility Data	Outcome
	French)			and had at least one ANC visit to a health facility		
14.	Gabrysch et al., 2011a	Zambia	*Ward (cluster)* level, GPS based	Population and Housing census in 2000	Health Facility Census in 2005	Proportions of population living within 15km of any type of delivery care and<br><br>within 15km of EmOC services.
15.	Gabrysch et al., 2011b	Rural Zambia	*Cluster* level: Linked by geographic coordinates to calculate straight-line distance	2007 Zambia Demographic Health Survey	Health Facility Census in 2005	Facility delivery
16.	Gage and Zomahoun, 2011	51 Local government areas in the Federal	*Local government area* level: administrative linking	Household survey of 2009 - end of COMPASS project survey	Health facility survey of 2009 - end of COMPASS project survey	FP knowledge and use

No.	Authors and Year	Study Site	Level of linking	Source and Timing of Household Data	Source and Timing of Facility Data	Outcome
		Capital Territory and the states of Bauchi, Kano, Lagos and Nasarawa in Nigeria				
17.	Gething et al., 2012	Ghana	*Household* level: journey time maps combined with population grid	Data on real journeys made by women in labor seeking care from IMMPACT project (for model calibration)<br><br>Core Welfare Indicator Questionnaire in 2003 (for mechanized transport estimation)	Authors developed a geospatial database of health facilities. Exact timing was not reported.	Journey-time for all women of childbearing age to their nearest health facility

No.	Authors and Year	Study Site	Level of linking	Source and Timing of Household Data	Source and Timing of Facility Data	Outcome
18.	Hong et al., 2006	Egypt	*Community* level: distances between communities and FP facilities	2003 DHS	2002 SPA	IUD use
19.	Hotchkiss et al., 2003	Morocco	*Cluster* level: women were linked to the nearest facility and three nearest private doctors within 30km	1995 DHS	1995 SAM	Use of maternal health care
20.	Hotchkiss, 1998	Phillipines (Cebu)	*Household* level: straight line distance in meters between women and health facilities	Cebu Longitudinal Health and Nutrition Study: 3,118 women with non-missing baseline and birth information surveyed. Timing was not reported.	Supplementary Health Facility survey of 82 public and private facilities. Timing was not reported.	Choice of delivery alternative
21.	Hounton et al., 2008	Burkina Faso (Ouargaye and Diapaga districts)	*Village* level: a health center is administratively assigned by the government to each village	Household census in 2006 that asked about childbirths in 2001-2006	Survey of all 43 health facilities in 2006	Utilization of delivery care
22.	Hutchinson and	South Africa (Eastern	*Household* level: each household was linked to the	2002/3 Primary Health Care Evaluation	2002 Facility Survey of 210 government	Use of VCT

No.	Authors and Year	Study Site	Level of linking	Source and Timing of Household Data	Source and Timing of Facility Data	Outcome
	Mahlalela, 2006	Cape)	closest facility	Survey of 3,374 adults	facilities selected based on proximity to communities in household survey	
23.	Kante and Pison, 2010 (in French)	Senegal (Bandafassi)	*Household* level: each household was linked to a specific hospital and a specific health facility	Survey of women who gave birth in the last 12 months in 2007	Physical accessibility only	Use of ANC
24.	Katende et al., 2003	Delivery of Improved Services for Health (DISH) Project areas in Uganda (12 districts)	*Cluster* level: households were linked to facilities located within the cluster or surrounding rings of cluster	1999 DISH Evaluation Survey (Household Questionnaire of 1,766 women aged 15-49)	1999 DISH Evaluation Survey (General Facility Survey of 292 facilities that served sampled population)	FP
25.	Khan et al., 2006	Tanzania	*Cluster* level: households were linked to facilities serving the cluster	1996 DHS	1996 Service Availability Survey	ANC, delivery, child immunization
26.	Kruk et	Liberia (Nimba	*Household* level: households were linked with	Surveys of 1,434	Survey of 43 facilities	Access to treatment of

No.	Authors and Year	Study Site	Level of linking	Source and Timing of Household Data	Source and Timing of Facility Data	Outcome
	al., 2010	county)	the nearest facility using travel time and straight-line distance	households in 2008	in 2008	malaria, HIV testing and counseling, obstetric care, IMCI, and mental health care
27.	Kyei et al., 2012	Zambia	*Cluster* level: straight-line distance between cluster and the nearest facility within 10km	2007 DHS	2005 Health Facility Census	Use of antenatal care
28.	Lohela et al., 2012	Malawi and Zambia	*Cluster* level: straight-line distance between cluster and the nearest facility	2004 Malawi Demographic Health Survey ; 2007 Zambia Demographic Health Survey	2002 Malawi National Health Facility Census; 2005 Zambia National Health Facility Census	Early neonatal mortality,<br>Facility use for delivery
29.	Magnani et al., 1999	Morocco	*Household* level: distance between household and the nearest FP facility within 30 km	1995 Panel DHS	1995 Service Availability Module (SAM)	Contraceptive intentions and use
30.	Malqvist	Quang Ninh	*Household* level: distance	Survey of mothers of	Physical accessibility	Neonatal

No.	Authors and Year	Study Site	Level of linking	Source and Timing of Household Data	Source and Timing of Facility Data	Outcome
	et al., 2010	province, Vietnam	between household and the nearest facility	197 neonatal deaths and 686 referents in 2008-2009.	only.	mortality
31.	Marchant et al., 2008	Tanzania	*Cluster* level	Household survey across 21 districts in 2005, 2006, and 2007.	Survey of facility serving each cluster in 2005, 2006, and 2007.	Malaria prevention during pregnancy
32.	Marchant and Schellenberg, 2013	Ethiopia, Nigeria and Uttar Pradesh, India	*Cluster* level	Survey in 2012 of women who gave birth in the last 12 months	Surveys of facilities and community level health volunteers (front line health workers) also in 2012	Skilled birth attendance
33.	Marchant et al., 2015	Ethiopia, Nigeria and Uttar Pradesh, India	*Cluster* level	Survey in 2012 of women who gave birth in the last 12 months	Surveys of facilities and community level health volunteers (front line health workers) also in 2012	ANC, prevention of hemorrhage during skilled birth attendance, post-partum and post-natal care
34.	MEASUR	Bangladesh	*Household* level: women	Community survey as	Facility survey as part	FP, ANC,

No.	Authors and Year	Study Site	Level of linking	Source and Timing of Household Data	Source and Timing of Facility Data	Outcome
	E Evaluation , 2003	(rural areas in 5 divisions)	were linked with the nearest static and satellite clinics	part of the project's evaluation in 2001	of the project's evaluation in 2001	receipt of 2+ tetatus toxoid vaccinations
35.	Mensch et al., 1996	Peru	*Cluster* level	1991-92 DHS	1992 Situation Analysis (of facilities)	Contraceptive use
36.	Micah et al., 2014	Uganda (rural)	*Local council (administrative)* level: household was linked to the most used health facility in each local council area	Uganda National Panel Survey 2010-2012 (Household Module)	Uganda National Panel Survey 2010-2012 (Community Module)	Use of formal medical care
37.	Mroz et al., 1999	Tanzania	*Cluster* level	1991-92 DHS	1993 Tanzania Accessibility Survey and 1991-92 DHS facility module	Contraceptive use
38.	Mwabu et al., 1993	Meru district, Kenya	Not described	Survey of 315 households in 1980-1981.	Survey of 15 government and private facilities in 1980-1981.	Choice of medical treatment
39.	Mwaliko et al.,	Webuye health and	*Household* level: Euclidean distance from households to	Census sampling of 13,333 households in	Physical accessibility only.	Any delivery at home in the last

No.	Authors and Year	Study Site	Level of linking	Source and Timing of Household Data	Source and Timing of Facility Data	Outcome
	2014	demographic surveillance system in Bungoma County, Kenya	health facilities	2008-2009		year
40.	Pullum, 1991	Guatemala (rural areas)	*Cluster* level	1987 Demographic Health Survey-Individual module	1987 Demographic Health Survey-Service Availability Module (SAM)	Current use of contraception
41.	Rose et al., 1999	10 countries (Benin, Central African Republic, Mali, Uganda, Zimbabwe, Bangladesh, the Philippines, Indonesia,	*Cluster* level (rural only)	DHS between 1993 and 1996/7	Service Availability Module (SAM) in the same time period	Use of maternity services

No.	Authors and Year	Study Site	Level of linking	Source and Timing of Household Data	Source and Timing of Facility Data	Outcome
		Bolivia and Haiti)				
42.	Seiber and Bertrand, 2002	Guatemala	*Cluster* level: mean distance between cluster and facilities	1995/96 DHS (1190 ladino and 1825 Mayan women)	1997 Provider Survey (296 facilities linked to respondents)	FP use
43.	Seiber et al., 2005	Guatemala	*Cluster* level	1995/6 Guatemala DHS (1345 Guatemalan women)	1997 Providers Census	FP, intensity of MCH use<br>(index for intensity of ANC, postnatal care, delivery attendance and immunization)
44.	Sia et al., 2007 (in French)	Burkina Faso (rural)	*Cluster* level: each household was linked with aggregated measures of service environment	1998 DHS	1997 Health Ministry Statistical Yearbook	Child immunization
45.	Skiles et	Rwanda (rural areas	*Cluster* level with 4 geographic techniques: (i)	Rwanda Interim Demographic and	2007 Rwanda Service	For 3 linking methods:

No.	Authors and Year	Study Site	Level of linking	Source and Timing of Household Data	Source and Timing of Facility Data	Outcome
	al., 2013	only)	administrative boundary link; (ii) Euclidean buffer link; (iii) road network link; (iv) Kernel density estimation link	Health Survey in 2007-2008	Provision Assessment	distance to nearest health facility, number of health facilities linked to the cluster, type of linked facilities, FP methods available in at least one facility linked to the cluster, HIV services available in at least one facility linked to the cluster.<br><br>For Kernel Density Estimation (KDE) link: FP readiness and

No.	Authors and Year	Study Site	Level of linking	Source and Timing of Household<br><br>Data	Source and Timing of Facility Data	Outcome
						VCT readiness
46.	Steele et al., 1999	Morocco	*Cluster* level	1995 DHS	1992 SAM	Contraceptive dynamics (discontinuation and switching)
47.	Tanser et al., 2006	South Africa (rural KwaZulu-Natal only)	*Household* level: walking time and travel time with public transport	Survey of 23,000 households. Timing was not reported.	Physical accessibility only.	Use of primary health care
48.	Ukwuani et al., 2006	Uganda and Tanzania	*Cluster* level	DHS in 1995-96	1999 Tanzania Community Survey<br><br>(Uganda DHS already collected data at the community level)	Condom use
49.	Wang et al., 2012	Four East African Countries (Kenya, Rwanda, Uganda,	*Regional* level	Kenya: 2003 DHS, 2008-09 DHS<br><br>Uganda: 2006 DHS<br><br>Rwanda: 2007-08	Kenya: 2004 SPA, 2010 SPA<br><br>Uganda: 2007 SPA<br><br>Rwanda: 2007 SPA	Women's current use of any modern contraceptive method

No.	Authors and Year	Study Site	Level of linking	Source and Timing of Household Data	Source and Timing of Facility Data	Outcome
		Tanzania)		DHS<br>Tanzania: 2004-05 DHS	Tanzania: 2006 SPA	
50.	Wang et al., 2014	Haiti	*Cluster* level	2012 Demographic and Health Survey	2013 Service Provision Assessment	Facility based delivery
51.	Wang et al., 2015	Haiti and Malawi	*Cluster* level: straight-line distance using GPS coordinates	Haiti DHS 2012<br>Malawi DHS 2010	SPAs 2013-14	FP, ANC, HIV testing, child health, and malaria
Direct Linking (Exact Matching)						
1.	Akin and Guilkey, 1995	Nigeria<br>(Ogun State)	*Individual* level: patients randomly selected at facilities	Household survey of 100 patients. Timing was not reported.	Survey of 86 health facilities in public sector. Timing was not reported.	Demand for outpatient health care
2.	Arifeen et al., 2004	Bangladesh (Matlab district)	*Individual* level: patients identified from facility records	Survey at the home of children <5 in 2000.	Survey and observations of 20 first level outpatient facilities in the district in 2000.	Use of health facility and correct treatment for sick children

No.	Authors and Year	Study Site	Level of linking	Source and Timing of Household Data	Source and Timing of Facility Data	Outcome
3.	Feikin et al., 2009	Kenya (Asembo)	*Individual* level: each child under 5 was linked to a clinic visit	Demographic surveillance data of children <5 collected in 2003-2004	Each child was linked to a clinic visit in 2003-2004	Use of pediatric health care
4.	Kruger et al., 2013	Southern edge of Mbulu district in Manyara region in northern Tanzania	*Individual* level: patients identified from facility records	Retrospectively collected vaccination data of 3,868 infants from RCH clinic records in 1998, 1999, 2006 and 2007	Eight mobile RCH clinics located in the Iraqw or Datoga mainland in 1998-1999	Number of vaccinations per registered infant during the first year of life
5.	Nesbitt et al., 2013	Ghana (Brong Ahafo)	*Individual* level: facility reported by mothers	Surveillance data of women of childbearing age in the demographic surveillance district collected in 2010.	Health facility assessment of 86 facilities in the surveillance area in 2010.	Intrapartum and postnatal care
6.	Seclen-Palacin et al., 2003	Peru (34 health districts hospitals and	*Individual* level: facility reported by mothers	Interviews on knowledge (including alarm obstetric signs) and attitudes with 143	Quality of service provision assessment conducted in 2000 and evaluating 5 aspects of	Contribution of quality improvement program on

No.	Authors and Year	Study Site	Level of linking	Source and Timing of Household Data	Source and Timing of Facility Data	Outcome
	(in Spanish)	health centers)		users, after ANC visits in 2000	MNCH service at 23 hospitals, 33 Health Centers, and 18 Health posts	knowledge and attitudes towards usage of MCH (antenatal care) services
7.	Soubeiga, 2012 (in French)	Burkina Faso	*Individual* level: women identified from facility records	Survey (in 2009) of women who gave birth in January – March 2009	Survey of 30 health facilities in two districts (rural only) in 2009	Use of ANC services and childbirth preparations
8.	Tumlimson and Speizer, 2013	Kenya (urban)	*Individual* level: facility reported by mothers	2010 baseline survey of n=8,170 women from 5 urban areas	2011 survey of 260 facilities	FP

Note:
[1] Haversine formula: calculates straight-line distance where the earth is assumed to be a perfect sphere and has radius of 6,378.1km.
[2] Each province in the DRC is divided into health zones, which are further divided into health areas. Each health zone is comprised of one general reference hospital and is tasked with serving anywhere from 100,000 people in rural areas to 150,000 people in urban areas. Each health area is comprised of at least one health center and serves on average 17 villages in rural areas or neighborhoods in urban areas.
